# Supplementary material for: Genome-wide association mapping for component traits of drought tolerance in dry beans (Phaseolus vulgaris L.)
Source: PLoS One. 2023 May 18;18(5):e0278500. doi: 10.1371/journal.pone.0278500 (PMC10194967; doi:10.1371/journal.pone.0278500)
Supplement: S1 Table — (DOCX) [file pone.0278500.s001.docx]

**S1 Table Characteristics of bean genotypes used in the study, their sources and structure membership coefficient (K2) for K = 2.**

| **Type** | **Genotype** | **Code** | **Genepool** | **Seed Size** | **Market Class** | **Growth habit** | **K2** | **Source** |
| --- | --- | --- | --- | --- | --- | --- | --- | --- |
| Cultivar | PROTEA | G1 | M | Small | Small white | Indeterminate | 0.00 – 1.00 | CBI Zimbabwe |
| Cultivar | SMC16 | G2 | ADM | Medium | White | Determinate | 0.14 – 0.86 | CBI Zimbabwe |
| Breeding line | RAZ 42 | G3 | M | Small | Small white | Determinate | 0.01 – 0.99 | CBI Zimbabwe |
| Landrace | BIOFORT SMALL SEEDED 15 | G4 | M | Small | Small white | Determinate | 0.00 – 1.00 | CBI Zimbabwe |
| Breeding line | SAA12 | G5 | A | Large | Large white | Determinate | 1.00 – 0.00 | CBI Zimbabwe |
| Breeding line | SAA2 | G6 | A | Large | Large white | Determinate | 0.99 – 0.01 | CBI Zimbabwe |
| Breeding line | NAE80 | G7 | M | Small | Small white | Determinate | 0.00 – 1.00 | ABC Colombia |
| Breeding line | NAE13 | G8 | M | Small | Small white | Determinate | 0.00 – 1.00 | ABC Colombia |
| Breeding line | ZABRA16575-73F22 | G9 | M | Small | Small white | Indeterminate | 0.00 – 1.00 | ABC Colombia |
| Breeding line | SAA18 | G10 | A | Large | Large white | Determinate | 0.99 – 0.01 | ABC Colombia |
| Breeding line | ZABR-16576-20F22 | G11 | M | Small | Small white | Indeterminate | 0.00 – 1.00 | ABC Colombia |
| Breeding line | G48 | G12 | M | Small | Small white | Determinate | 0.00 – 1.00 | ABC Colombia |
| Landrace | CZ108-52 | G13 | M | Medium | White | Determinate | 0.08 – 0.92 | ABC Colombia |
| Breeding line | SMC21 | G14 | ADM | Medium | White | Determinate | 0.12 – 0.88 | ABC Colombia |
| Breeding line | RAZ11 | G15 | M | Small | Small white | Determinate | 0.01 – 0.99 | ABC Colombia |
| Landrace | SWAT-10 (SELIAM 10) | G16 | M | Small | Small white | Determinate | 0.00 – 1.00 | ABC Colombia |
| Breeding line | SAA1 | G17 | A | Large | Large | Determinate | 0.99 – 0.01 | ABC Colombia |
| Breeding line | RAZ-36 | G18 | M | Small | Small white | Determinate | 0.00 – 1.00 | ABC Colombia |
| Breeding line | SAB-662 | G19 | M | Large | Large white | Determinate | 0.03 – 0.97 | ABC Colombia |
| Breeding line | NAVY LINE-48 | G20 | M | Small | Small white | Indeterminate | 0.00 – 1.00 | ABC Colombia |
| Breeding line | CIM-NAV02-17-3 | G21 | M | Small | Small white | Indeterminate | 0.00 – 1.00 | ABC Colombia |
| Breeding line | G738 | G22 | M | Small | Small white | Determinate | 0.00 – 1.00 | ABC Colombia |
| Breeding line | G49 | G23 | M | Small | Small white | Determinate | 0.00 – 1.00 | ABC Colombia |
| Landrace | CZ108-53 | G24 | M | Medium | White | Determinate | 0.03 – 0.97 | ABC Colombia |
| Landrace | RWR2154 | G25 | ADM | Medium | White | Determinate | 0.14 – 0.86 | ABC Colombia |
| Landrace | CAB 2 | G26 | M | Medium | White | Indeterminate | 0.06 – 0.94 | ABC Colombia |
| Landrace | SWAT-12 (SELIAM-11) | G27 | M | Small | Small white | Determinate | 0.00 – 1.00 | ABC Colombia |
| Breeding line | G54 | G28 | M | Small | Small white | Determinate | 0.00 – 1.00 | ABC Colombia |
| Breeding line | ZABRA16575-57F22 | G29 | M | Small | Small white | Indeterminate | 0.01 – 0.99 | ABC Colombia |
| Breeding line | ZABRA16575-26F22 | G30 | M | Small | Small white | Indeterminate | 0.00 – 1.00 | ABC Colombia |
| Breeding line | G30 | G31 | M | Small | Small white | Determinate | 0.00 – 1.00 | ABC Colombia |
| Breeding line | NAVY LINE-60 | G32 | M | Small | Small white | Indeterminate | 0.00 – 1.00 | ABC Colombia |
| Breeding line | SAA17 | G33 | A | Large | Large white | Determinate | 1.00 – 0.00 | ABC Colombia |

**S1 Table (Continued).**

| **Type** | **Genotype** | **Code** | **Genepool** | **Seed Size** | **Market Class** | **Growth habit** | **K2** | **Source** |
| --- | --- | --- | --- | --- | --- | --- | --- | --- |
| Breeding line | NAVY19 | G34 | M | Small | Small white | Indeterminate | 0.00 – 1.00 | ABC Colombia |
| Breeding line | G70 | G35 | M | Small | Small white | Determinate | 0.00 – 1.00 | ABC Colombia |
| Breeding line | SMB31 | G36 | M | Medium | White | Determinate | 0.03 – 0.97 | ABC Colombia |
| Cultivar | SMC17 | G37 | M | Medium | White | Determinate | 0.09 – 0.91 | ABC Colombia |
| Breeding line | G53 | G38 | M | Small | Small white | Determinate | 0.00 – 1.00 | ABC Colombia |
| Landrace | R02/1 | G39 | M | Small | Small white | Determinate | 0.09 – 0.92 | ABC Colombia |
| Breeding line | G14 | G40 | M | Small | Small white | Determinate | 0.00 – 1.00 | ABC Colombia |
| Breeding line | DAB562 | G41 | A | Large | Sugar | Determinate | 0.92 – 0.08 | ABC Colombia |
| Breeding line | NAE24 | G42 | M | Small | Small white | Determinate | 0.00 – 1.00 | ABC Colombia |
| Breeding line | ICABUNSIxSXB405/9C-1C-1C-3 | G43 | M | Small | Small white | Indeterminate | 0.00 – 1.00 | ABC Colombia |
| Breeding line | G99 | G44 | M | Small | Small white | Determinate | 0.00 – 1.00 | ABC Colombia |
| Breeding line | SAA7 | G45 | A | Large | Large | Determinate | 1.00 – 0.00 | ABC Colombia |
| Cultivar | UBR(92)25 | G46 | M | Small | Small white | Determinate | 0.00 – 1.00 | ABC Colombia |
| Breeding line | G40 | G47 | M | Small | Small white | Determinate | 0.00 – 1.00 | ABC Colombia |
| Breeding line | NAE60 | G48 | M | Small | Small white | Determinate | 0.04 – 0.96 | ABC Colombia |
| Landrace | Michigan Pea Bean | G49 | M | Small | Small white | Indeterminate | 0.02 – 0.98 | ABC Colombia |
| Landrace | Chore | G50 | M | Small | Small white | Indeterminate | 0.03 – 0.97 | ABC Colombia |
| Landrace | BASABEER | G51 | M | Small | Small white | Indeterminate | 0.09 – 0.91 | ABC Colombia |
| Cultivar | AWASH-1 | G52 | M | Small | Small white | Indeterminate | 0.00 – 1.00 | ABC Colombia |
| Breeding line | ICA BUNSIxSXB405-1C-1C | G53 | M | Small | Small white | Indeterminate | 0.00 – 1.00 | ABC Colombia |
| Landrace | NAIN DEKYONDO | G54 | M | Small | Small white | Indeterminate | 0.00 – 1.00 | ABC Colombia |
| Breeding line | ZABRA16575-51F22 | G55 | M | Small | Small white | Indeterminate | 0.00 – 1.00 | ABC Colombia |
| Breeding line | G6 | G56 | M | Small | Small white | Determinate | 0.00 – 1.00 | ABC Colombia |
| Breeding line | NAVY46 | G57 | M | Small | Small white | Indeterminate | 0.05 – 0.95 | ABC Colombia |
| Breeding line | G16 | G58 | M | Small | Small white | Determinate | 0.04 – 0.96 | ABC Colombia |
| Breeding line | G37 | G59 | M | Small | Small white | Determinate | 0.00 – 1.00 | ABC Colombia |
| Breeding line | NAE19 | G60 | M | Small | Small white | Determinate | 0.00 – 1.00 | ABC Colombia |
| Landrace | Chercher | G61 | M | Small | Small white | Indeterminate | 0.04 – 0.96 | ABC Colombia |
| Landrace | Argene | G62 | M | Small | Small white | Indeterminate | 0.00 – 1.00 | ABC Colombia |
| Landrace | SIRAJ | G63 | M | Small | Small white | Indeterminate | 0.08 – 0.92 | ABC Colombia |
| Breeding line | NAE40 | G64 | M | Small | Small white | Determinate | 0.00 – 1.00 | ABC Colombia |
| Breeding line | G550 | G65 | A | Large | Sugar | Determinate | 0.96 – 0.04 | ABC Colombia |
| Landrace | CZ108-27 | G66 | M | Medium | White | Determinate | 0.00 – 1.00 | ABC Colombia |
| Breeding line | NAE70 | G67 | M | Large | Small white | Determinate | 0.00 – 1.00 | ABC Colombia |

**S1 Table (Continued).**

| **No.** | **Genotype** | **Code** | **Gene pool** | **Seed Size** | **Market Class** | **Growth habit** | **K2** | **Source** |
| --- | --- | --- | --- | --- | --- | --- | --- | --- |
| Breeding line | G24 | G68 | M | Small | Small white | Determinate | 0.00 – 1.00 | ABC Colombia |
| Breeding line | G90 | G69 | M | Small | Small white | Determinate | 0.00 – 1.00 | ABC Colombia |
| Breeding line | SAA19 | G70 | A | Large | Large white | Determinate | 0.98 – 0.02 | ABC Colombia |
| Breeding line | G34 | G71 | M | Small | Small white | Determinate | 0.00 – 1.00 | ABC Colombia |
| Breeding line | NAE87 | G72 | M | Small | Small white | Determinate | 0.00 – 1.00 | ABC Colombia |
| Cultivar | AWASH MELKA | G73 | M | Small | Small white | Indeterminate | 0.00 – 1.00 | ABC Colombia |
| Landrace | NAZARETHE2 | G74 | M | Small | Small white | Indeterminate | 0.03 – 0.97 | ABC Colombia |
| Landrace | SAB792 | G75 | ADM | Large | Large white | Determinate | 0.86 – 0.14 | ABC Colombia |
| Landrace | CZ113-13 | G76 | A | Medium | White | Determinate | 0.95 – 0.05 | ABC Colombia |
| Breeding line | SAB793 | G77 | A | Large | Large white | Indeterminate | 0.93 – 0.07 | ABC Colombia |
| Breeding line | ICA BUNSIxSXB405/3C-1C-1C-8 | G78 | M | Small | Small white | Indeterminate | 0.00 – 1.00 | ABC Colombia |
| Breeding line | NAVY LINE 22 | G79 | ADM | Medium | White | Indeterminate | 0.15 – 0.85 | ABC Colombia |
| Breeding line | G100 | G80 | M | Small | Small white | Determinate | 0.00 – 1.00 | ABC Colombia |
| Breeding line | SAB791 | G81 | A | Large | Large white | Determinate | 0.88 – 0.12 | ABC Colombia |
| Breeding line | G27 | G82 | M | Small | Small white | Determinate | 0.00 – 1.00 | ABC Colombia |
| Breeding line | NAE78 | G83 | M | Small | Small white | Determinate | 0.00 – 1.00 | ABC Colombia |
| Cultivar | AWASH 2 | G84 | M | Small | Small white | Indeterminate | 0.00 – 1.00 | ABC Colombia |
| Landrace | SWAT-09 (SELIAM 9) | G85 | M | Small | Small white | Indeterminate | 0.00 – 1.00 | ABC Colombia |
| Breeding line | ZABRA16573-25F22 | G86 | M | Small | Small white | Indeterminate | 0.00 – 1.00 | ABC Colombia |
| Breeding line | ICABUNSIxSXB405/4C-1C-1C-8 | G87 | M | Small | Small white | Indeterminate | 0.00 – 1.00 | ABC Colombia |
| Breeding line | RAZ-44 | G88 | M | Small | Small white | Determinate | 0.03 – 0.97 | ABC Colombia |
| Breeding line | G32 | G89 | M | Small | Small white | Determinate | 0.00 – 1.00 | ABC Colombia |
| Breeding line | CIM-NAV02-35-1 | G90 | M | Small | Small white | Indeterminate | 0.05 – 0.95 | ABC Colombia |
| Cultivar | CANPSULA | G91 | M | Small | Small white | Indeterminate | 0.00 – 1.00 | ABC Colombia |
| Breeding line | CIM-NAV02-10-1 | G92 | M | Small | Small white | Indeterminate | 0.04 – 0.96 | ABC Colombia |
| Breeding line | CIM-DWRF-CLIM01-1-1 | G93 | M | Small | Small white | Indeterminate | 0.00 – 1.00 | ABC Malawi |
| Landrace | MUTWAKIL | G94 | M | Small | Small white | Determinate | 0.08 – 0.92 | ABC Malawi |
| Breeding line | MAB89 | G95 | A | Large | Red | Determinate | 0.98 – 0.02 | ABC Malawi |
| Breeding line | CIM-RM02-71-1 | G96 | A | Large | Red mottled | Indeterminate | 0.96 – 0.04 | ABC Malawi |
| Cultivar | G97 (Seed-co) | G97 | A | Large | Sugar | Determinate | 0.92 – 0.08 | ABC Malawi |
| Breeding line | ZABRA16575-86F22 | G98 | A | Large | White | Indeterminate | 1.00 – 0.00 | ABC Malawi |
| Breeding line | DAB363 | G99 | A | Medium | Sugar | Determinate | 1.00 – 0.00 | ABC Malawi |
| Breeding line | DAB367 | G100 | A | Medium | Sugar | Determinate | 0.97 – 0.03 | ABC Malawi |

**S1 Table (Continued).**

| **No.** | **Genotype** | **Code** | **Gene pool** | **Seed Size** | **Market Class** | **Growth habit** | **K2** | **Source** |
| --- | --- | --- | --- | --- | --- | --- | --- | --- |
| Breeding line | DAB482 | G101 | A | Medium | Sugar | Determinate | 0.99 – 0.01 | ABC Malawi |
| Cultivar | Sweet Violet | G102 | A | Large | Sugar | Indeterminate | 0.98 – 0.02 | ABC Malawi |
| Landrace | CZ104-65 | G103 | A | Medium | White | Determinate | 0.98 – 0.02 | ABC Malawi |
| Breeding line | DAB210 | G104 | A | Large | Sugar | Determinate | 1.00 – 0.00 | ABC Malawi |
| Breeding line | DAB470 | G105 | M | Small | Sugar | Determinate | 0.00 – 1.00 | ABC Malawi |
| Breeding line | G19 | G106 | A | Medium | White | Determinate | 0.98 – 0.02 | ABC Malawi |
| Breeding line | VTTT926/9-6 | G107 | A | Large | Sugar | Indeterminate | 0.94 – 0.06 | ABC Malawi |
| Breeding line | DAB142 | G108 | ADM | Medium | Sugar | Determinate | 0.89 – 0.11 | ABC Malawi |
| Cultivar | NUA674 | G109 | ADM | Medium | Sugar | Determinate | 0.11 – 0.89 | ABC Malawi |
| Landrace | Waju | G110 | A | Medium | White | Determinate | 0.96 – 0.04 | ABC Malawi |
| Breeding line | DAB447 | G111 | A | Medium | Sugar | Determinate | 0.93 – 0.07 | ABC Malawi |
| Breeding line | DAB62 | G112 | A | Medium | Sugar | Determinate | 0.93 – 0.07 | ABC Malawi |
| Cultivar | Gloria | G113 | A | Medium | Sugar | Indeterminate | 1.00 – 0.00 | ABC Malawi |
| Breeding line | CIM-RM09-ALS-BSM-11 | G114 | ADM | Large | Red mottled | Indeterminate | 0.82 – 0.18 | ABC Malawi |
| Landrace | CZ104-11 | G115 | A | Medium | White | Determinate | 1.00 – 0.00 | ABC Malawi |
| Breeding line | DAB133 | G116 | M | Small | Sugar | Determinate | 0.07 – 0.93 | ABC Malawi |
| Breeding line | G22 | G117 | A | Medium | white | Determinate | 1.00 – 0.00 | ABC Malawi |
| Breeding line | CIM-RM00-321LN02 | G118 | A | Large | Red mottled | Indeterminate | 0.99 – 0.01 | ABC Malawi |
| Breeding line | VTTT925/2-5-2-2 | G119 | A | Large | Sugar | Indeterminate | 0.93 – 0.07 | ABC Malawi |
| Breeding line | CIM-CBB-FeZn08-21-2 | G120 | M | Small | Red mottled | Indeterminate | 0.00 – 1.00 | ABC Malawi |
| Breeding line | ZABRA16573-78F22 | G121 | A | Medium | White | Indeterminate | 1.00 – 0.00 | ABC Malawi |
| Breeding line | CIM-RM09-ALS-BSM-1 | G122 | A | Large | Red mottled | Indeterminate | 1.00 – 0.00 | ABC Malawi |
| Breeding line | DAB361 | G123 | A | Medium | Sugar | Determinate | 1.00 – 0.00 | ABC Malawi |
| Breeding line | DAB487 | G124 | A | Medium | Sugar | Determinate | 1.00 – 0.00 | ABC Malawi |
| Breeding line | CIM-RM09-ALS-BSM-12 | G125 | A | Large | Red mottled | Indeterminate | 1.00 – 0.00 | ABC Malawi |
| Breeding line | CIM-RM09-ALS-BSM-14 | G126 | A | Large | Red mottled | Indeterminate | 0.95 – 0.05 | ABC Malawi |
| Breeding line | CIM-SUG07-ALS-2 | G127 | A | Medium | Sugar | Determinate | 1.00 – 0.00 | ABC Malawi |
| Breeding line | DAB224 | G128 | M | Medium | Sugar | Determinate | 0.00 – 1.00 | ABC Malawi |
| Breeding line | G75 | G129 | ADM | Small | Small white | Determinate | 0.68 – 0.32 | ABC Malawi |
| Breeding line | DAB115 | G130 | ADM | Medium | Sugar | Determinate | 0.86 – 0.14 | ABC Malawi |
| Breeding line | DAB296 | G131 | M | Medium | Sugar | Determinate | 0.02 – 0.98 | ABC Malawi |
| Breeding line | RAZ-34 | G132 | A | Medium | White | Determinate | 1.00 – 0.00 | ABC Malawi |
| Breeding line | DAB355 | G133 | A | Medium | Sugar | Determinate | 1.00 – 0.00 | ABC Malawi |

**S1 Table (Continued).**

| **No.** | **Genotype** | **Code** | **Gene pool** | **Seed Size** | **Market Class** | **Growth habit** | **K2** | **Source** |
| --- | --- | --- | --- | --- | --- | --- | --- | --- |
| Breeding line | DAB368 | G134 | A | Medium | Sugar | Determinate | 1.00 – 0.00 | ABC Malawi |
| Breeding line | DAB539 | G135 | A | Medium | Sugar | Determinate | 0.99 – 0.01 | ABC Malawi |
| Breeding line | DAB63 | G136 | ADM | Medium | Sugar | Determinate | 0.69 – 0.31 | ABC Malawi |
| Breeding line | DAB477 | G137 | ADM | Medium | Sugar | Determinate | 0.79 – 0.21 | ABC Malawi |
| Breeding line | CZ104-72 | G138 | A | Medium | White | Determinate | 1.00 – 0.00 | ABC Malawi |
| Breeding line | CZ113-15 | G139 | M | Small | White | Determinate | 0.00 – 1.00 | ABC Malawi |
| Breeding line | G97 | G140 | A | Medium | White | Determinate | 1.00 – 0.00 | ABC Malawi |
| Breeding line | CIM-RM02-36-1 | G141 | ADM | Large | Red mottled | Indeterminate | 0.13 – 0.88 | ABC Malawi |
| Breeding line | RCB234 | G142 | A | Medium | Red | Determinate | 0.98 – 0.02 | ABC Malawi |
| Breeding line | NUA59-4 | G143 | ADM | Large | Red mottled | Determinate | 0.73 – 0.27 | ABC Malawi |
| Breeding line | NAVY LINE-47 | G144 | A | Medium | White | Determinate | 1.00 – 0.00 | ABC Malawi |
| Landrace | RWR222 | G145 | A | Medium | Red mottled | Determinate | 0.93 – 0.07 | ABC Malawi |
| Cultivar | AFR703 | G146 | A | Large | Red kidney | Determinate | 0.98 – 0.02 | ABC Malawi |
| Breeding line | CIM-SUG07-ALS-S1-3 | G147 | A | Medium | Sugar | Determinate | 0.98 – 0.02 | ABC Malawi |
| Breeding line | CIM-RM-03-03-45 | G148 | A | Large | Red mottled | Determinate | 0.94 – 0.06 | ABC Malawi |
| Breeding line | DAB523 | G149 | A | Medium | Sugar | Determinate | 0.92 – 0.08 | ABC Malawi |
| Breeding line | CIM-DRHT-SUG-S5-3 | G150 | A | Medium | Sugar | Determinate | 0.96 – 0.04 | ABC Malawi |
| Breeding line | CIM-RK05-ALS-39 | G151 | M | Medium | Red kidney | Determinate | 0.00 – 1.00 | ABC Malawi |
| Breeding line | ICN BunsixSxB405/7C-1C-1C-5 | G152 | A | Medium | White | Indeterminate | 1.00 – 0.00 | ABC Malawi |
| Breeding line | DAB124 | G153 | A | Medium | Sugar | Determinate | 0.96 – 0.04 | ABC Malawi |
| Breeding line | CIM-RM02-134-1 | G154 | ADM | Large | Red mottled | Determinate | 0.79 – 0.21 | ABC Malawi |
| Breeding line | NUA735-2 | G155 | M | Medium | Red mottled | Determinate | 0.02 – 0.98 | ABC Malawi |
| Breeding line | NAVY LINE-52 | G156 | A | Medium | White | Indeterminate | 1.00 – 0.00 | ABC Malawi |
| Breeding line | DCIM-RM09-ALSBSM- | G157 | A | Large | Red mottled | Determinate | 1.00 – 0.00 | ABC Malawi |
| Cultivar | Sweet William | G158 | ADM | Medium | Sugar | Determinate | 0.88 – 0.12 | ABC Malawi |
| Cultivar | G159 (Seed-Co) | G159 | A | Medium | Sugar | Determinate | 1.00 – 0.00 | ABC Malawi |
| Breeding line | DAB299 | G160 | A | Medium | Sugar | Determinate | 0.98 – 0.02 | ABC Uganda |
| Breeding line | DAB378 | G161 | A | Medium | Sugar | Determinate | 1.00 – 0.00 | ABC Uganda |
| Breeding line | DAB150 | G162 | A | Medium | Sugar | Determinate | 0.90 – 0.10 | ABC Uganda |
| Breeding line | DAB112 | G163 | M | Medium | Sugar | Determinate | 0.00 – 1.00 | ABC Uganda |
| Breeding line | ZABRA16575-60F22 | G164 | ADM | Small | White | Indeterminate | 0.79 – 0.21 | ABC Uganda |
| Breeding line | DAB143 | G165 | M | Medium | Sugar | Determinate | 0.00 – 1.00 | ABC Uganda |
| Cultivar | G166 (Seed-Co) | G166 | M | Medium | Sugar | Determinate | 0.00 – 1.00 | ABC Uganda |

**S1 Table (Continued).**

| **No.** | **Genotype** | **Code** | **Gene pool** | **Seed Size** | **Market Class** | **Growth habit** | **K2** | **Source** |
| --- | --- | --- | --- | --- | --- | --- | --- | --- |
| Breeding line | NAVY LINE-54 | G167 | A | Medium | White | Indeterminate | 1.00 – 0.00 | ABC Uganda |
| Breeding line | DAB410 | G168 | A | Medium | Sugar | Determinate | 1.00 – 0.00 | ABC Uganda |
| Cultivar | Cherry | G169 | A | Medium | Red mottled | Determinate | 0.93 – 0.07 | ABC Uganda |
| Breeding line | CIM-SUG05-01-02 | G170 | A | Medium | Sugar | Determinate | 0.90 – 0.10 | ABC Uganda |
| Breeding line | DAB360 | G171 | A | Medium | Sugar | Determinate | 1.00 – 0.00 | ABC Uganda |
| Breeding line | KG27-8 | G172 | A | Medium | Red mottled | Determinate | 1.00 – 0.00 | ABC Uganda |
| Breeding line | CZ104-61 | G173 | A | Medium | White | Determinate | 0.98 – 0.02 | ABC Uganda |
| Breeding line | CIM-RM00-104 | G174 | M | Large | Red mottled | Determinate | 0.08 – 0.92 | ABC Uganda |
| Breeding line | ZABRA16574-37F22 | G175 | A | Medium | White | Indeterminate | 1.00 – 0.00 | ABC Uganda |
| Breeding line | DAB302 | G176 | A | Medium | Sugar | Determinate | 0.97 – 0.03 | ABC Uganda |
| Cultivar | G177 (Seed-co) | G177 | M | Medium | Sugar | Determinate | 0.02 – 0.98 | ABC Uganda |
| Breeding line | CIM-NAV08-1 | G178 | A | Medium | White | Indeterminate | 0.93 – 0.07 | ABC Colombia |
| Breeding line | GLP585/MLB49-89A-3 | G179 | ADM | Medium | Red mottled | Indeterminate | 0.80 – 0.20 | EIAR Ethiopia |
| Breeding line | DAB78 | G180 | A | Medium | Sugar | Determinate | 1.00 – 0.00 | EIAR Ethiopia |
| Breeding line | DAB61 | G181 | A | Medium | Sugar | Determinate | 0.98 – 0.02 | EIAR Ethiopia |
| Breeding line | CIM-SUG07-ALS-S1-3 | G182 | ADM | Medium | Sugar | Determinate | 0.63 – 0.37 | ABC Malawi |
| Breeding line | CIM-SUG02-14-3 | G183 | A | Medium | Sugar | Determinate | 0.94 – 0.06 | ABC Malawi |
| Breeding line | DAB91 | G184 | A | Medium | Sugar | Determinate | 0.99 – 0.01 | ABC Malawi |
| Breeding line | DAB433 | G185 | A | Medium | Sugar | Determinate | 1.00 – 0.00 | ABC Malawi |

K2 = structure membership coefficient/ancestry probability, ADM = admixed, M = Mesoamerican, A = Andean, *CBI* Crop Breeding Institute, ABC = Alliance of Bioversity International and International Centre for Tropical Agriculture, EIAR = Ethiopian Institute of Agricultural Research. Admixed includes genotypes that are 10 to 90% Andean or Mesoamerican according to the structure analysis results.
