# Supplementary material for: Genome-wide association mapping for component traits of drought tolerance in dry beans (Phaseolus vulgaris L.)
Source: PLoS One. 2023 May 18;18(5):e0278500. doi: 10.1371/journal.pone.0278500 (PMC10194967; doi:10.1371/journal.pone.0278500)
Supplement: S2 Table — (DOCX) [file pone.0278500.s002.docx]

**S4 Table Drought tolerance indices and predicted genotype values for grain yield (across environments) of the 185 Andean-Mesoamerican diversity panel.**

| **Genotype** | **GYD (kg/ha)** | **DSI** | **GMP** | **DTI** | **%GYR** | **Mean rank** |
| --- | --- | --- | --- | --- | --- | --- |
| G1 | 242.60 | 1.84 | 167.50 | 0.03 | 55.07 | 172.50 |
| G2 | 400.90 | 1.73 | 349.90 | 0.12 | 51.96 | 162.50 |
| G3 | 570.80 | -2.41 | 557.20 | 0.35 | -72.21 | 61.75 |
| G4 | 1191.40 | 1.50 | 1132.70 | 1.50 | 44.96 | 89.75 |
| G5 | 483.10 | -1.74 | 426.70 | 0.20 | -52.34 | 83.25 |
| G6 | 508.30 | 0.50 | 503.30 | 0.26 | 15.11 | 99.00 |
| G7 | 586.30 | -2.12 | 570.80 | 0.35 | -63.69 | 61.50 |
| G8 | 506.00 | -2.95 | 481.90 | 0.24 | -88.46 | 73.75 |
| G9 | 765.30 | 1.54 | 722.00 | 0.53 | 46.23 | 116.00 |
| G10 | 488.40 | -0.75 | 485.00 | 0.23 | -22.57 | 84.00 |
| G11 | 424.10 | 2.34 | 356.40 | 0.14 | 70.26 | 171.50 |
| G12 | 160.90 | 2.26 | 134.20 | 0.02 | 67.90 | 180.50 |
| G13 | 612.50 | 1.74 | 569.40 | 0.33 | 52.24 | 136.75 |
| G14 | 305.30 | -1.64 | 279.80 | 0.08 | -49.34 | 95.50 |
| G15 | 519.40 | 0.47 | 515.20 | 0.31 | 14.01 | 92.25 |
| G16 | 842.60 | -2.90 | 704.10 | 0.65 | -86.87 | 45.00 |
| G17 | 507.40 | 0.53 | 500.10 | 0.24 | 15.75 | 102.25 |
| G18 | 549.30 | -0.22 | 530.80 | 0.28 | -6.63 | 79.50 |
| G19 | 585.20 | 1.25 | 543.90 | 0.30 | 37.39 | 122.50 |
| G20 | 676.20 | 1.98 | 607.80 | 0.40 | 59.43 | 135.00 |
| G21 | 555.80 | 1.19 | 526.20 | 0.27 | 35.78 | 123.50 |
| G22 | 548.40 | 0.90 | 539.80 | 0.30 | 27.02 | 106.75 |
| G23 | 506.70 | 0.44 | 505.10 | 0.25 | 13.06 | 96.25 |
| G24 | 553.70 | 1.11 | 522.00 | 0.28 | 33.34 | 118.75 |
| G25 | 391.90 | 0.97 | 373.60 | 0.14 | 29.12 | 131.25 |
| G26 | 350.50 | 0.89 | 343.70 | 0.14 | 26.55 | 128.75 |
| G27 | 813.30 | -7.68 | 733.10 | 0.63 | -230.5 | 42.00 |
| G28 | 560.00 | 0.68 | 554.20 | 0.30 | 20.37 | 97.50 |
| G29 | 535.00 | 2.00 | 472.80 | 0.23 | 60.13 | 156.50 |
| G30 | 751.20 | 1.58 | 703.60 | 0.49 | 47.46 | 120.25 |
| G31 | 873.80 | 1.09 | 833.30 | 0.69 | 32.82 | 90.00 |
| G32 | 781.30 | 2.06 | 619.10 | 0.38 | 61.83 | 137.75 |
| G33 | 253.70 | -0.89 | 236.40 | 0.06 | -26.70 | 101.00 |
| G34 | 558.60 | 0.05 | 545.00 | 0.30 | 1.56 | 82.00 |
| G35 | 170.80 | 0.72 | 157.70 | 0.03 | 21.75 | 131.00 |
| G36 | 365.50 | 1.24 | 347.00 | 0.12 | 37.10 | 146.50 |
| G37 | 294.20 | 1.56 | 280.70 | 0.09 | 46.82 | 160.25 |
| G38 | 619.70 | 0.51 | 615.30 | 0.40 | 15.35 | 85.00 |
| G39 | 1015.40 | 0.78 | 936.60 | 1.09 | 23.39 | 68.75 |
| G40 | 541.70 | 0.53 | 536.30 | 0.29 | 15.88 | 96.00 |
| G41 | 541.90 | 0.82 | 508.50 | 0.25 | 24.67 | 109.75 |
| G42 | 482.60 | 0.01 | 478.90 | 0.24 | 0.26 | 92.75 |
| G43 | 600.70 | -0.60 | 585.70 | 0.38 | -17.93 | 68.75 |
| G44 | 499.50 | -0.25 | 490.90 | 0.23 | -7.47 | 86.50 |
| G45 | 294.40 | 1.12 | 287.60 | 0.10 | 33.55 | 142.50 |
| G46 | 380.10 | 0.96 | 341.90 | 0.12 | 28.84 | 134.75 |
| G47 | 60.60 | -0.54 | 58.80 | 0.01 | -16.30 | 107.50 |
| G48 | 407.20 | 2.13 | 352.10 | 0.13 | 63.80 | 169.75 |
| G49 | 47.20 | 0.42 | 45.40 | 0.00 | 12.50 | 122.50 |
| G50 | 466.00 | -1.74 | 443.90 | 0.21 | -52.25 | 81.50 |
| G51 | 796.30 | 1.64 | 748.10 | 0.56 | 49.35 | 120.75 |
| G52 | 513.40 | 0.94 | 505.80 | 0.25 | 28.19 | 115.00 |

**S4 Table (Continued).**

| **Genotype** | **GYD (kg/ha)** | **DSI** | **GMP** | **DTI** | **%GYR** | **Mean rank** |
| --- | --- | --- | --- | --- | --- | --- |
| G53 | 554.20 | 0.80 | 541.60 | 0.29 | 24.01 | 104.00 |
| G54 | 426.90 | -0.03 | 426.10 | 0.19 | -0.80 | 97.50 |
| G55 | 480.80 | 1.34 | 464.80 | 0.21 | 40.20 | 139.75 |
| G56 | 497.20 | -5.52 | 467.50 | 0.24 | -165.7 | 73.75 |
| G57 | 547.90 | -0.92 | 530.60 | 0.28 | -27.55 | 73.75 |
| G58 | 528.50 | 1.21 | 508.90 | 0.25 | 36.44 | 126.00 |
| G59 | 534.70 | 1.80 | 497.10 | 0.24 | 53.87 | 150.00 |
| G60 | 474.80 | 0.79 | 428.10 | 0.19 | 23.66 | 120.00 |
| G61 | 613.70 | 1.56 | 581.00 | 0.33 | 46.75 | 129.50 |
| G62 | 1109.60 | 0.30 | 1071.90 | 1.18 | 9.11 | 52.50 |
| G63 | 504.60 | 2.26 | 362.80 | 0.14 | 67.92 | 170.00 |
| G64 | 661.80 | -1.19 | 648.70 | 0.42 | -35.73 | 58.75 |
| G65 | 389.10 | 1.13 | 364.70 | 0.13 | 33.83 | 138.75 |
| G66 | 533.80 | 1.61 | 473.80 | 0.22 | 48.36 | 148.25 |
| G67 | 604.60 | -0.03 | 587.00 | 0.36 | -0.96 | 73.50 |
| G68 | 678.50 | 1.00 | 658.10 | 0.43 | 29.87 | 100.50 |
| G69 | 295.80 | 1.48 | 252.10 | 0.07 | 44.41 | 156.50 |
| G70 | 399.10 | 0.79 | 387.80 | 0.15 | 23.56 | 122.00 |
| G71 | 221.50 | -3.29 | 154.00 | 0.03 | -98.74 | 95.00 |
| G72 | 467.80 | 0.80 | 453.50 | 0.20 | 23.97 | 118.50 |
| G73 | 920.10 | 1.51 | 859.30 | 0.74 | 45.30 | 103.00 |
| G74 | 356.50 | 3.04 | 190.30 | 0.04 | 91.16 | 181.50 |
| G75 | 511.30 | -1.38 | 499.90 | 0.25 | -41.49 | 76.50 |
| G76 | 476.90 | 0.25 | 474.60 | 0.25 | 7.61 | 96.50 |
| G77 | 243.30 | 1.55 | 213.40 | 0.05 | 46.42 | 161.00 |
| G78 | 618.70 | -0.92 | 605.30 | 0.37 | -27.55 | 64.75 |
| G79 | 781.30 | -0.20 | 777.00 | 0.60 | -5.90 | 59.25 |
| G80 | 542.60 | -0.78 | 534.90 | 0.28 | -23.34 | 74.50 |
| G81 | 464.10 | 1.36 | 440.80 | 0.19 | 40.71 | 142.75 |
| G82 | 325.20 | 2.94 | 183.30 | 0.04 | 88.11 | 181.50 |
| G83 | 511.10 | 1.56 | 472.40 | 0.23 | 46.73 | 145.75 |
| G84 | 627.80 | -0.03 | 626.40 | 0.39 | -1.04 | 69.25 |
| G85 | 796.30 | 1.52 | 438.30 | 0.22 | 45.70 | 145.00 |
| G86 | 666.70 | 1.59 | 623.30 | 0.38 | 47.59 | 126.25 |
| G87 | 921.80 | -0.62 | 901.50 | 0.81 | -18.54 | 44.50 |
| G88 | 489.40 | 1.59 | 437.10 | 0.19 | 47.75 | 152.00 |
| G89 | 594.40 | 0.00 | 592.90 | 0.35 | 0.03 | 76.75 |
| G90 | 368.10 | 0.64 | 364.50 | 0.13 | 19.29 | 119.75 |
| G91 | 786.80 | 1.12 | 770.70 | 0.59 | 33.67 | 98.50 |
| G92 | 875.00 | 1.19 | 846.90 | 0.70 | 35.67 | 94.25 |
| G93 | 743.10 | 1.72 | 693.30 | 0.48 | 51.53 | 125.00 |
| G94 | 1203.70 | 1.19 | 1115.10 | 1.33 | 35.77 | 82.25 |
| G95 | 900.90 | 2.07 | 778.80 | 0.63 | 62.17 | 125.25 |
| G96 | 1040.70 | -0.90 | 1017.10 | 1.04 | -27.05 | 38.25 |
| G97 | 781.50 | 1.16 | 763.50 | 0.57 | 34.80 | 100.50 |
| G98 | 854.90 | 1.26 | 814.30 | 0.67 | 37.71 | 99.50 |
| G99 | 1465.00 | 0.17 | 1457.30 | 2.17 | 5.21 | 38.50 |
| G100 | 1158.80 | 1.64 | 1089.60 | 1.24 | 49.29 | 100.75 |
| G101 | 1964.80 | 1.32 | 1890.60 | 3.78 | 39.72 | 69.25 |
| G102 | 717.30 | 1.56 | 633.30 | 0.44 | 46.68 | 121.75 |
| G103 | 1463.00 | 0.94 | 1442.60 | 2.02 | 28.09 | 63.25 |
| G104 | 1608.80 | 0.58 | 1601.10 | 2.49 | 17.37 | 45.25 |
| G105 | 709.30 | 1.54 | 649.30 | 0.58 | 46.17 | 116.75 |
| G106 | 719.10 | -1.65 | 680.10 | 0.54 | -49.45 | 53.50 |
| G107 | 936.80 | 1.88 | 831.00 | 0.71 | 56.39 | 117.50 |

**S4 Table (Continued).**

| **Genotype** | **GYD (kg/ha)** | **DSI** | **GMP** | **DTI** | **%GYR** | **Mean rank** |
| --- | --- | --- | --- | --- | --- | --- |
| G108 | 1131.90 | -1.44 | 1105.10 | 1.19 | -43.32 | 32.00 |
| G109 | 697.20 | 2.11 | 504.70 | 0.32 | 63.31 | 148.50 |
| G110 | 921.30 | 1.43 | 796.20 | 0.71 | 42.92 | 104.00 |
| G111 | 1229.60 | 0.48 | 1193.80 | 1.40 | 14.53 | 53.00 |
| G112 | 1050.30 | 0.96 | 1024.20 | 1.04 | 28.90 | 76.25 |
| G113 | 1683.80 | 1.76 | 1548.00 | 2.35 | 52.77 | 91.50 |
| G114 | 1560.60 | 1.18 | 1518.90 | 2.28 | 35.28 | 70.50 |
| G115 | 1788.90 | 0.40 | 1765.40 | 3.04 | 11.87 | 35.50 |
| G116 | 796.30 | -0.50 | 785.40 | 0.62 | -15.14 | 55.00 |
| G117 | 1260.80 | -1.12 | 678.90 | 0.62 | -33.59 | 54.25 |
| G118 | 952.80 | 0.50 | 940.30 | 0.86 | 14.88 | 62.75 |
| G119 | 1180.10 | 0.85 | 1132.70 | 1.32 | 25.56 | 67.00 |
| G120 | 626.20 | -0.03 | 624.60 | 0.38 | -0.93 | 70.50 |
| G121 | 1114.20 | 0.47 | 972.70 | 0.96 | 13.98 | 59.50 |
| G122 | 1354.20 | 0.65 | 1329.00 | 1.86 | 19.59 | 55.00 |
| G123 | 1228.10 | 0.97 | 1207.10 | 1.42 | 28.95 | 70.00 |
| G124 | 1815.00 | -0.03 | 1805.50 | 3.19 | -0.78 | 26.00 |
| G125 | 1628.20 | -0.25 | 1590.20 | 2.49 | -7.39 | 26.75 |
| G126 | 1416.70 | 1.22 | 1370.70 | 1.83 | 36.52 | 78.75 |
| G127 | 1750.00 | -0.02 | 1733.00 | 2.94 | -0.73 | 29.00 |
| G128 | 808.30 | 2.21 | 556.30 | 0.46 | 66.16 | 140.75 |
| G129 | 853.40 | 2.42 | 418.10 | 0.21 | 72.67 | 167.75 |
| G130 | 987.30 | 0.20 | 970.90 | 0.92 | 6.09 | 54.25 |
| G131 | 1222.50 | 0.59 | 1195.30 | 1.40 | 17.63 | 56.75 |
| G132 | 433.60 | 2.15 | 313.50 | 0.11 | 64.64 | 173.5 |
| G133 | 1219.30 | 0.76 | 1195.10 | 1.40 | 22.91 | 61.25 |
| G134 | 892.60 | 0.91 | 873.80 | 0.74 | 27.41 | 79.50 |
| G135 | 1968.70 | -0.37 | 1956.90 | 3.75 | -11.17 | 19.75 |
| G136 | 1448.60 | 0.61 | 1440.50 | 2.01 | 18.26 | 52.00 |
| G137 | 1411.60 | 0.79 | 1395.50 | 1.89 | 23.56 | 57.50 |
| G138 | 1846.80 | 0.22 | 1826.10 | 3.25 | 6.50 | 31.00 |
| G139 | 833.10 | 1.63 | 756.60 | 0.57 | 49.03 | 119.50 |
| G140 | 986.10 | 1.74 | 912.10 | 1.02 | 52.23 | 108.25 |
| G141 | 1023.40 | -1.31 | 993.10 | 0.96 | -39.23 | 37.25 |
| G142 | 810.90 | 0.21 | 793.70 | 0.63 | 6.30 | 64.00 |
| G143 | 645.80 | 1.02 | 621.70 | 0.38 | 30.51 | 105.25 |
| G144 | 671.30 | 1.77 | 627.80 | 0.62 | 52.98 | 126.25 |
| G145 | 1184.60 | 1.34 | 1091.10 | 1.18 | 40.32 | 90.50 |
| G146 | 2067.40 | 1.18 | 1994.50 | 4.02 | 35.49 | 61.50 |
| G147 | 2080.10 | 1.26 | 1995.60 | 4.03 | 37.83 | 66.25 |
| G148 | 1418.10 | 1.00 | 1394.20 | 1.89 | 30.01 | 68.50 |
| G149 | 1607.90 | 0.48 | 1595.10 | 2.47 | 14.42 | 42.00 |
| G150 | 1758.80 | 0.40 | 1750.80 | 2.98 | 12.03 | 36.50 |
| G151 | 841.90 | 1.48 | 788.30 | 0.62 | 44.47 | 108.00 |
| G152 | 416.70 | 2.33 | 319.30 | 0.13 | 69.76 | 173.75 |
| G153 | 1017.40 | 0.75 | 1009.00 | 1.00 | 22.46 | 67.75 |
| G154 | 882.40 | 0.58 | 878.40 | 0.76 | 17.32 | 67.75 |
| G155 | 446.80 | 0.46 | 434.00 | 0.19 | 13.75 | 107.50 |
| G156 | 905.90 | -4.73 | 824.50 | 0.72 | -141.87 | 37.00 |
| G157 | 1308.00 | 1.38 | 1243.90 | 1.53 | 41.44 | 85.50 |
| G158 | 2017.10 | -0.01 | 1979.50 | 3.82 | -0.26 | 24.50 |
| G159 | 1743.30 | 1.12 | 1694.70 | 2.90 | 33.67 | 65.25 |
| G160 | 1246.80 | 0.92 | 1196.40 | 1.40 | 27.49 | 66.75 |
| G161 | 1195.40 | 0.85 | 1156.50 | 1.31 | 25.42 | 67.00 |
| G162 | 1828.70 | 0.57 | 1814.30 | 3.20 | 16.97 | 40.50 |

**S4 Table (Continued).**

| **Genotype** | **GYD (kg/ha)** | **DSI** | **GMP** | **DTI** | **%GYR** | **Mean rank** |
| --- | --- | --- | --- | --- | --- | --- |
| G163 | 997.70 | 1.98 | 784.00 | 0.61 | 59.50 | 124.50 |
| G164 | 518.50 | -9.82 | 389.30 | 0.17 | -294.46 | 79.50 |
| G165 | 761.60 | 1.65 | 720.30 | 0.54 | 49.49 | 122.50 |
| G166 | 815.30 | 0.93 | 804.50 | 0.63 | 27.82 | 85.25 |
| G167 | 620.40 | -12.98 | 495.40 | 0.28 | -389.46 | 66.25 |
| G168 | 1207.90 | 1.84 | 1083.40 | 1.24 | 55.10 | 106.50 |
| G169 | 629.40 | 1.26 | 599.10 | 0.35 | 37.76 | 118.50 |
| G170 | 1599.50 | 1.31 | 1543.20 | 2.34 | 39.22 | 76.50 |
| G171 | 1497.20 | 0.15 | 1490.20 | 2.17 | 4.63 | 37.00 |
| G172 | 1367.60 | 1.08 | 1287.00 | 1.63 | 32.45 | 72.00 |
| G173 | 1792.60 | 0.68 | 1780.40 | 3.07 | 20.40 | 46.00 |
| G174 | 982.40 | -6.11 | 900.20 | 0.90 | -183.33 | 31.75 |
| G175 | 407.40 | -2.00 | 315.70 | 0.12 | -59.87 | 91.50 |
| G176 | 2097.50 | 0.35 | 2084.00 | 4.25 | 10.60 | 29.50 |
| G177 | 813.40 | 0.92 | 802.70 | 0.63 | 27.70 | 85.25 |
| G178 | 887.30 | -4.17 | 855.80 | 1.03 | -125.00 | 33.00 |
| G179 | 1148.80 | 2.35 | 875.10 | 0.75 | 70.37 | 122.50 |
| G180 | 1838.90 | 0.09 | 1789.60 | 3.13 | 2.57 | 29.50 |
| G181 | 1614.10 | 1.03 | 1585.20 | 2.44 | 31.00 | 64.50 |
| G182 | 1415.30 | 1.21 | 1373.50 | 1.84 | 36.21 | 77.50 |
| G183 | 1540.30 | 0.97 | 1513.30 | 2.23 | 29.22 | 64.50 |
| G184 | 2222.70 | 0.16 | 2205.30 | 4.74 | 4.69 | 25.50 |
| G185 | 776.20 | 2.01 | 701.70 | 0.79 | 60.15 | 123.25 |

GYD = grain yield, DSI = drought susceptibility index, GMP = geometric mean productivity, DTI = drought tolerance index, %GYR = percent grain yield reduction. Note: Mean rank is the mean rank of a genotype across all the drought tolerance indices.
