# Supplementary material for: Genome-wide association mapping for component traits of drought tolerance in dry beans (Phaseolus vulgaris L.)
Source: PLoS One. 2023 May 18;18(5):e0278500. doi: 10.1371/journal.pone.0278500 (PMC10194967; doi:10.1371/journal.pone.0278500)
Supplement: S1 Fig — (DOCX) [file pone.0278500.s003.docx]

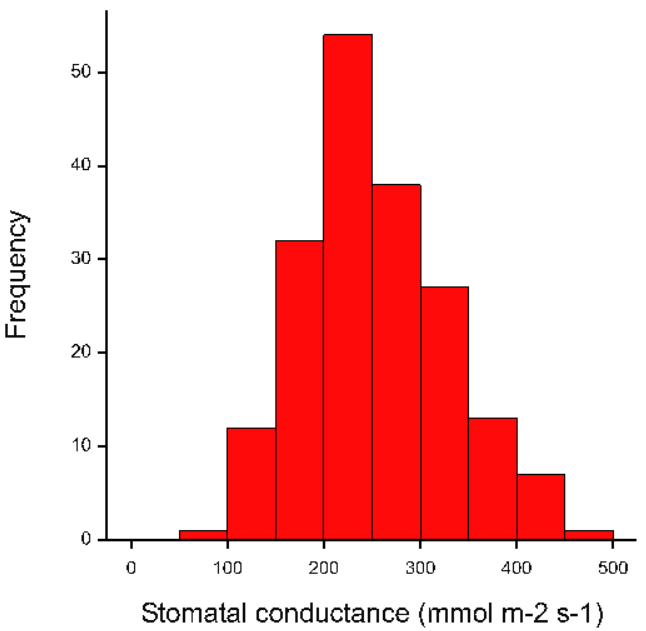


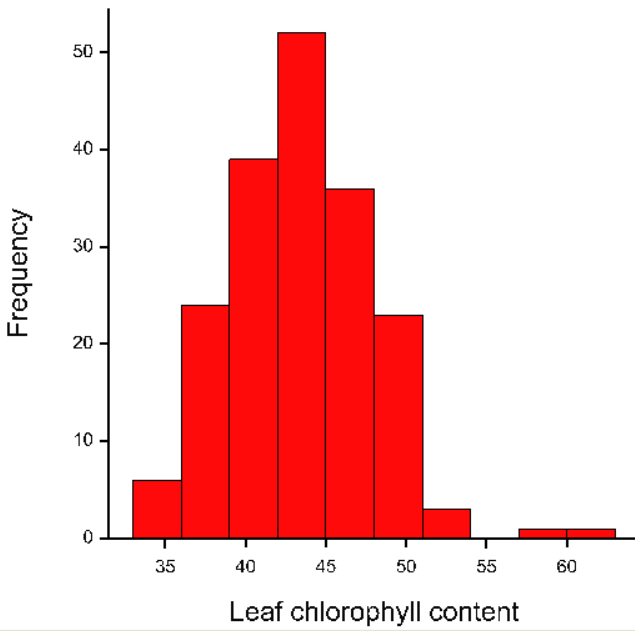


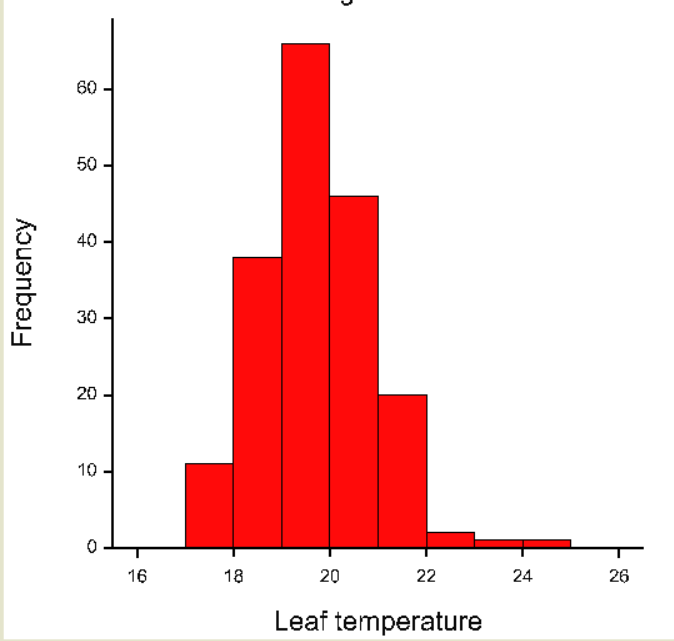


**Leaf temperature (℃)**


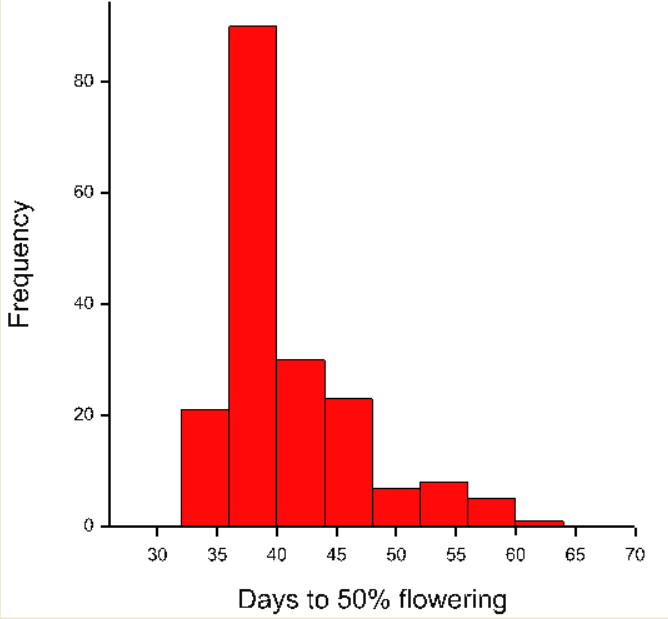


**S2 Fig. Frequency distribution of the studied agronomic and physiological traits observed under well-watered conditions in the andean-middle American diversity panel.**


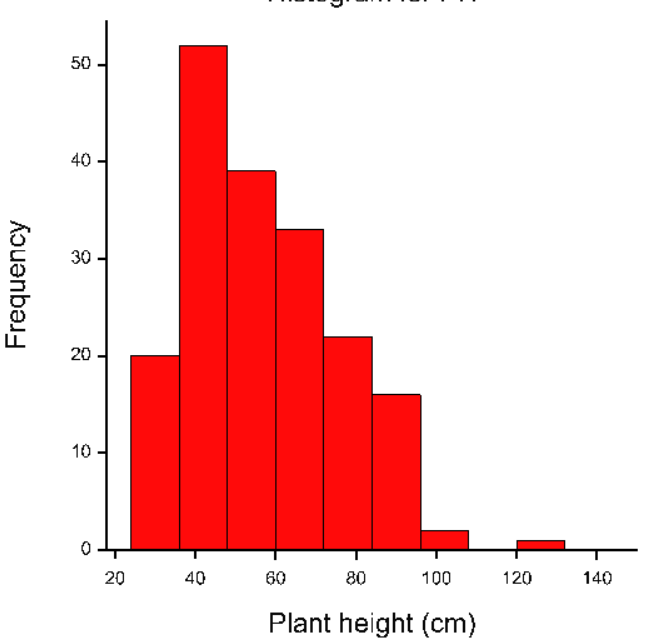


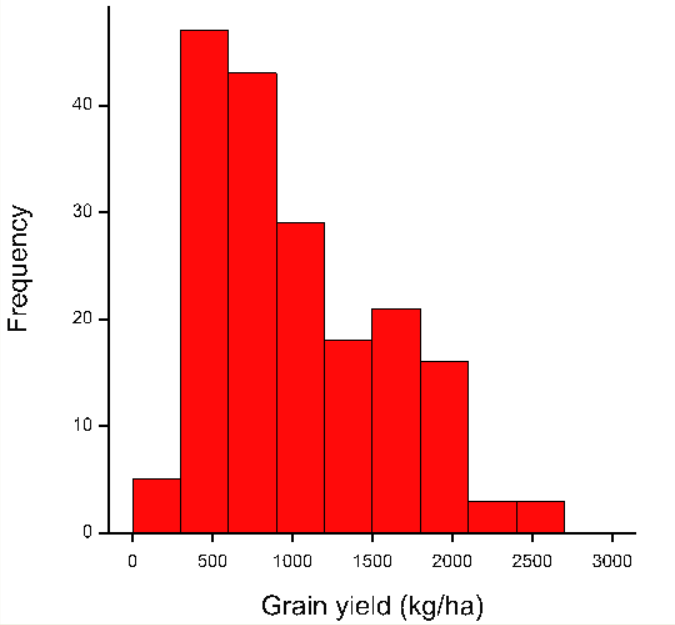


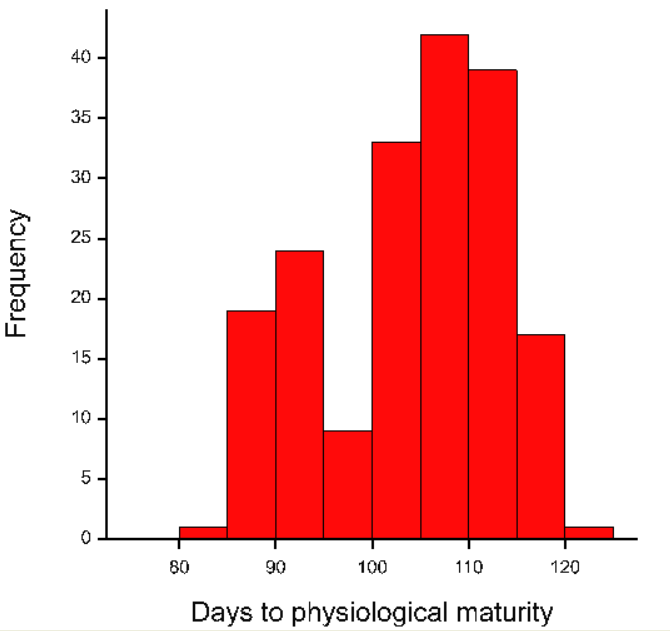


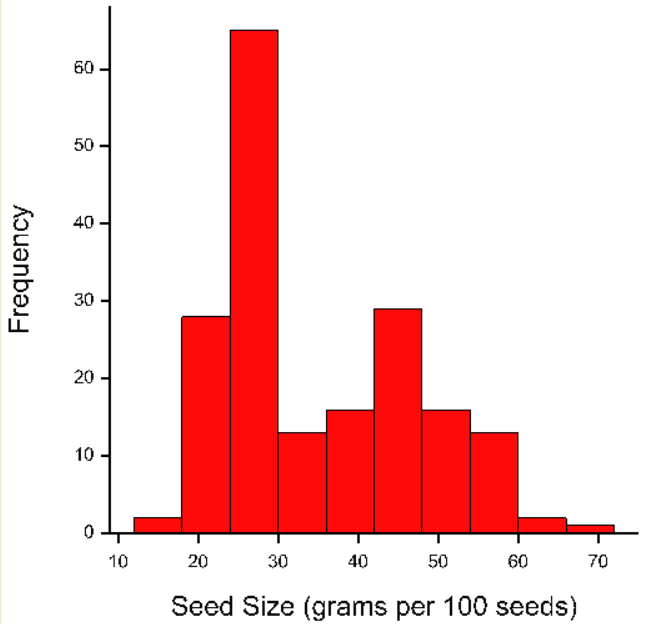


**S2 Fig (Continued).**
