# Supplementary material for: Genome-wide association mapping for component traits of drought tolerance in dry beans (Phaseolus vulgaris L.)
Source: PLoS One. 2023 May 18;18(5):e0278500. doi: 10.1371/journal.pone.0278500 (PMC10194967; doi:10.1371/journal.pone.0278500)
Supplement: S2 Fig — (DOCX) [file pone.0278500.s004.docx]

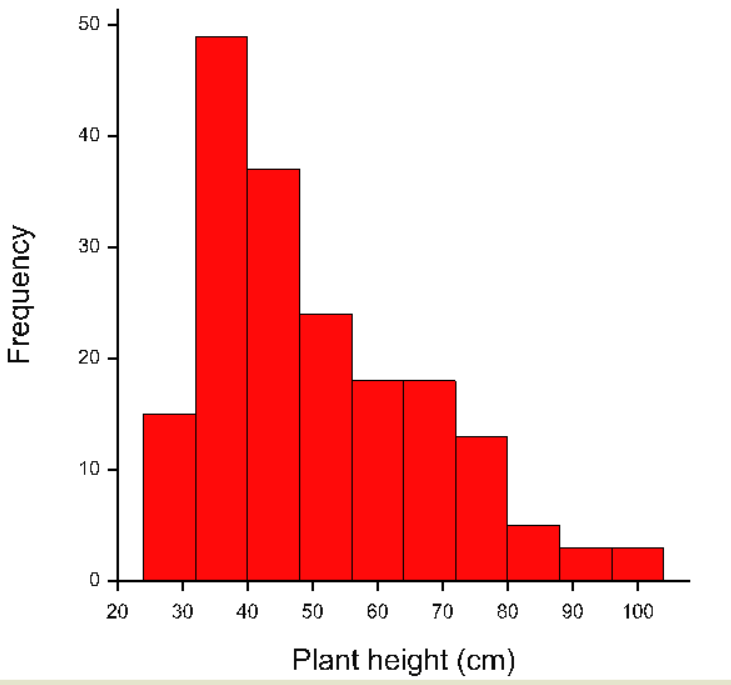


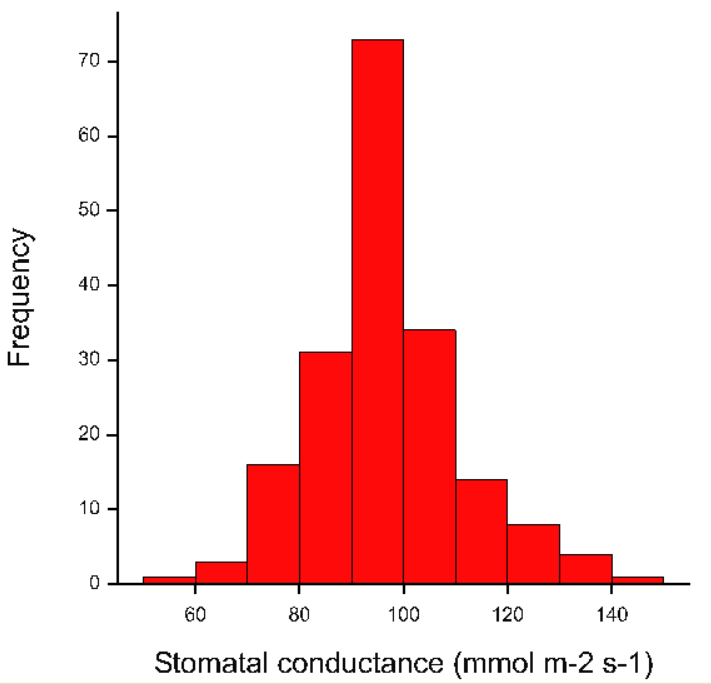


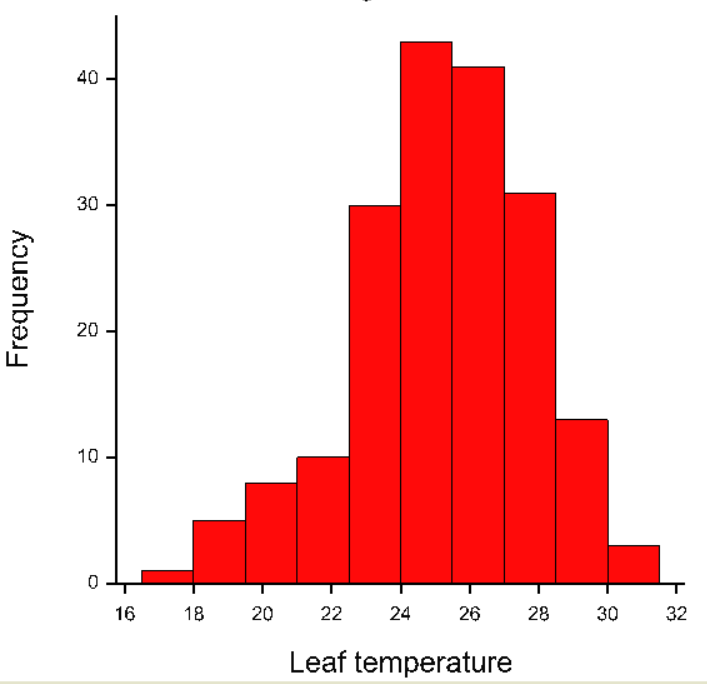


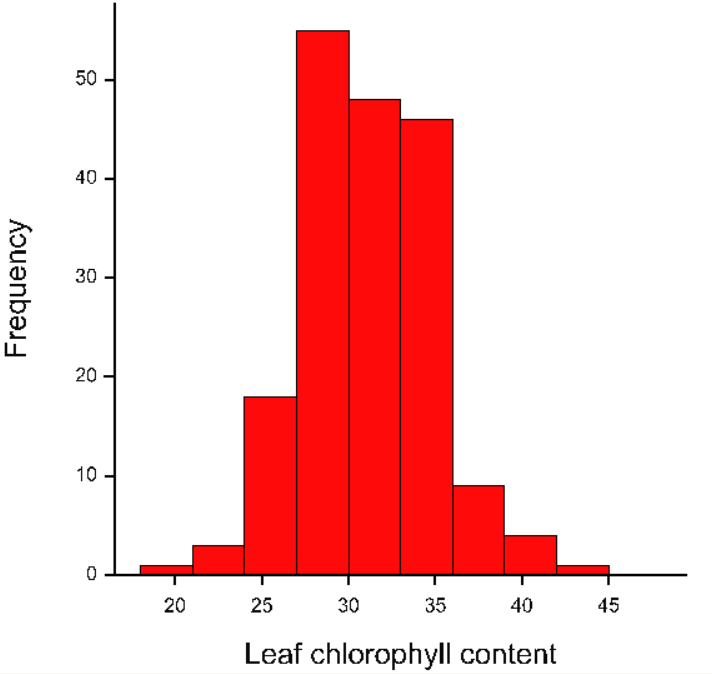


**Leaf temperature (℃)**

**S3 Fig. Frequency distribution of the studied agronomic and physiological traits observed under drought stressed conditions in the andean-middle American diversity panel.**


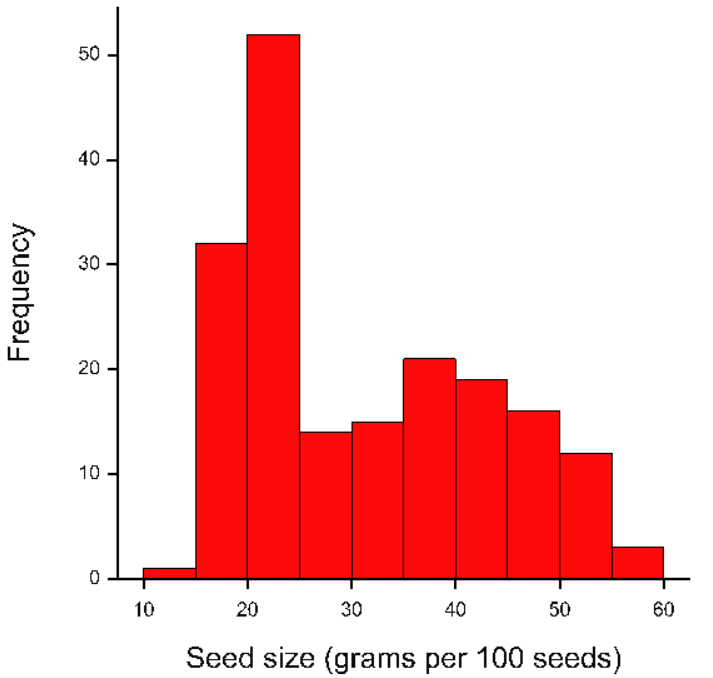


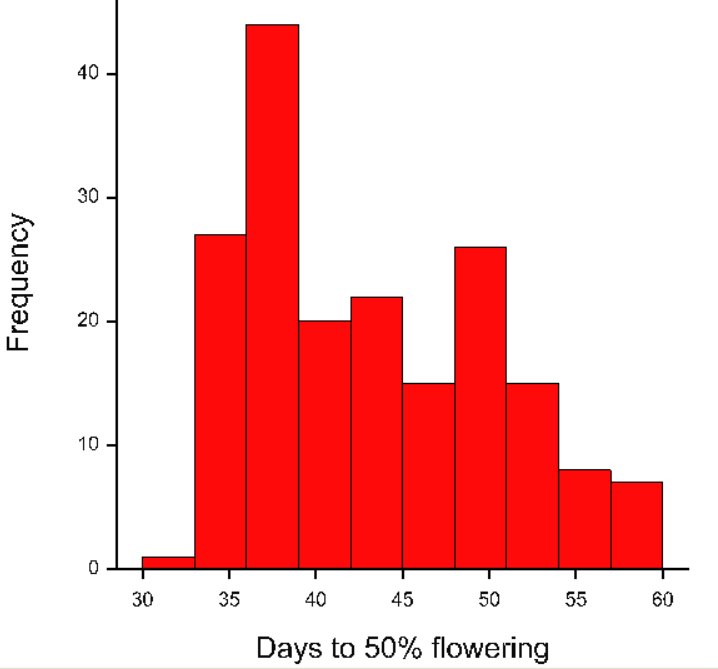


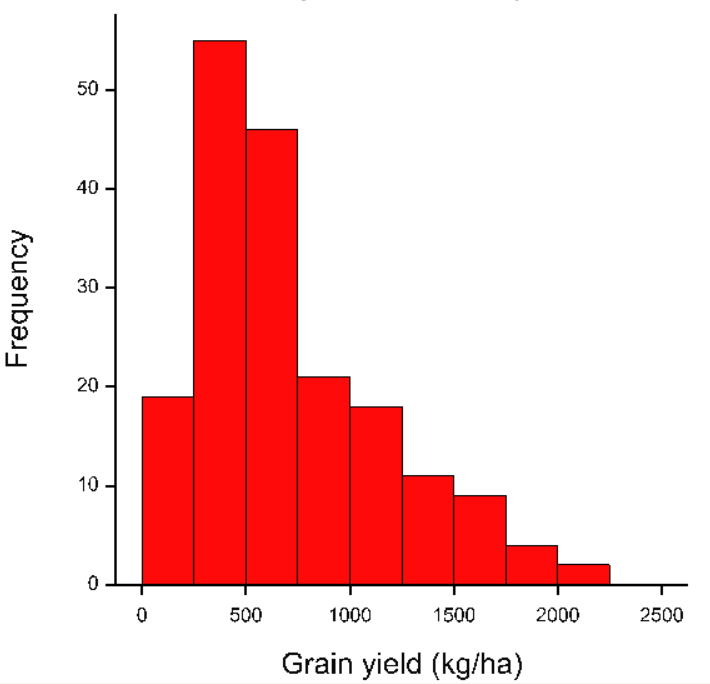


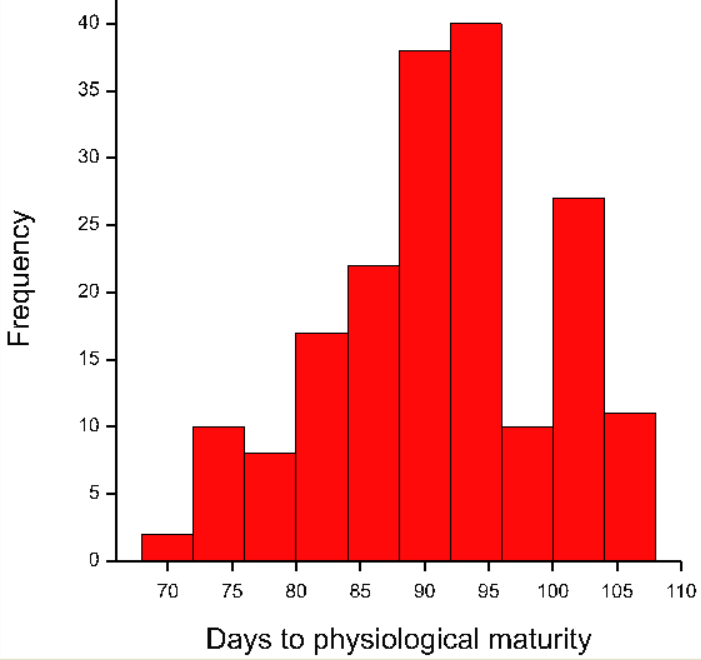


**S3 Fig (Continued).**
