# Supplementary material for: Genome-wide association mapping for component traits of drought tolerance in dry beans (Phaseolus vulgaris L.)
Source: PLoS One. 2023 May 18;18(5):e0278500. doi: 10.1371/journal.pone.0278500 (PMC10194967; doi:10.1371/journal.pone.0278500)
Supplement: S3 Fig — Note A = Leaf temperature, B = Days to 50% flowering, C = Grain yield, D = Plant height, E = Seed size, F = Stomatal conductance. (DOCX) [file pone.0278500.s005.docx]

**A**   **B**


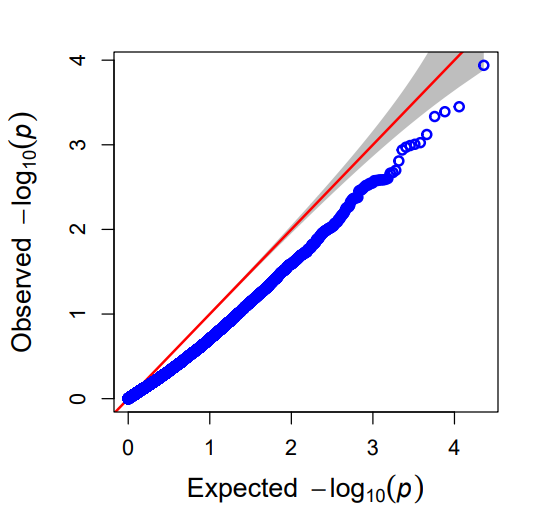


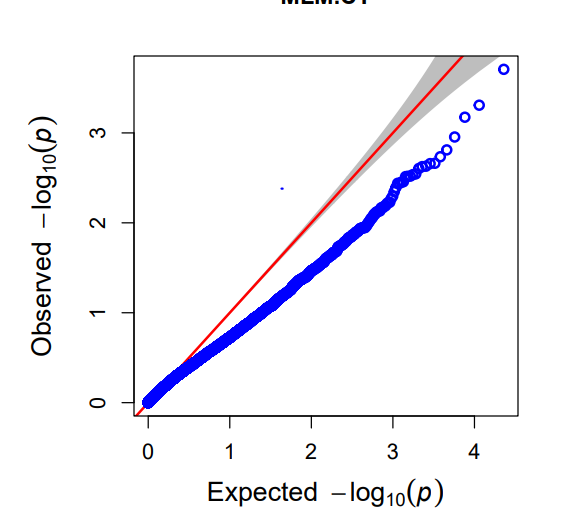


**C**  **D**


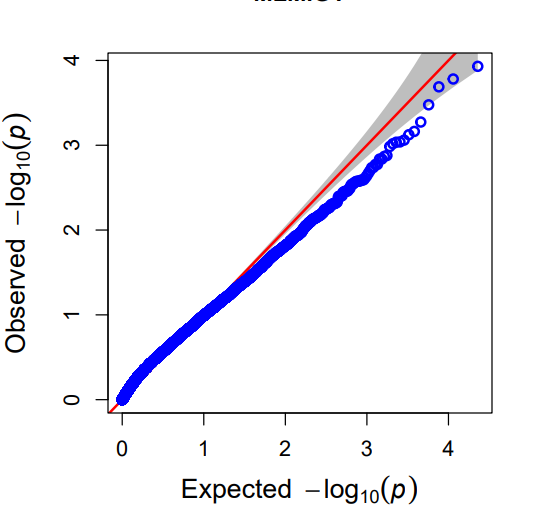


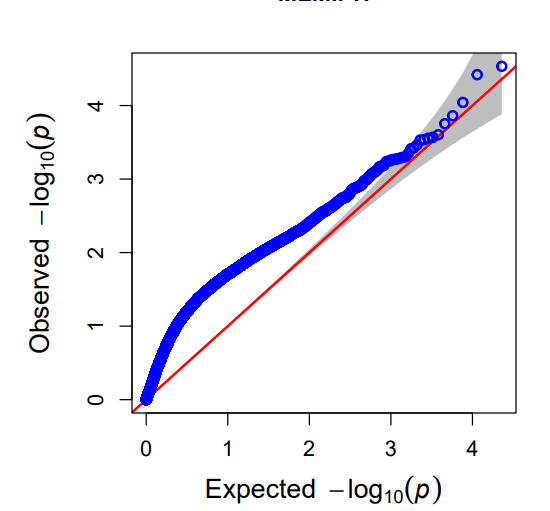


**E**  **F**


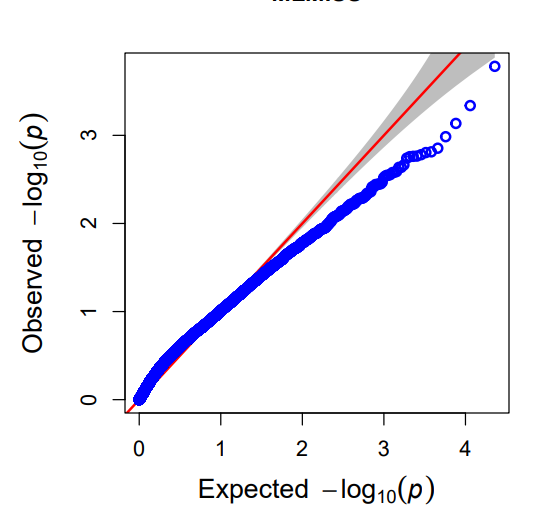


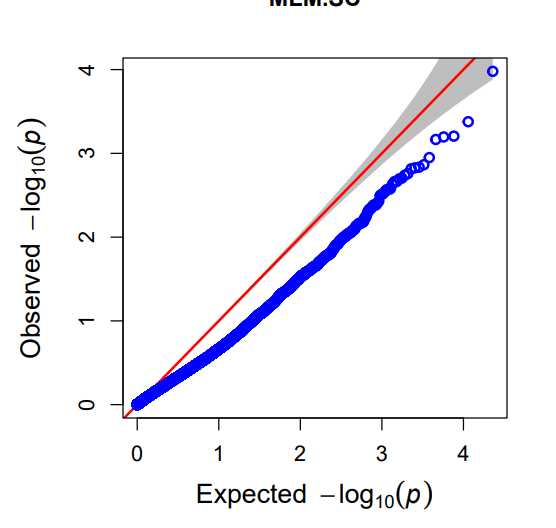


**S5 Fig. Quantile –Quantile (QQ) plots of the p- values observed and the expected from the genome-wide association study under drought stressed conditions.** Note A = Leaf temperature, B = Days to 50% flowering, C = Grain yield, D = Plant height, E = Seed size, F = Stomatal conductance.
