# Supplementary material for: Genome-wide association mapping for component traits of drought tolerance in dry beans (Phaseolus vulgaris L.)
Source: PLoS One. 2023 May 18;18(5):e0278500. doi: 10.1371/journal.pone.0278500 (PMC10194967; doi:10.1371/journal.pone.0278500)
Supplement: S4 Fig — Note A = Days to 50% flowering, B = Days to physiological maturity, C = Grain yield, D = Leaf chlorophyll content, E = Plant height, F = Leaf temperature. (DOCX) [file pone.0278500.s006.docx]

**A B**


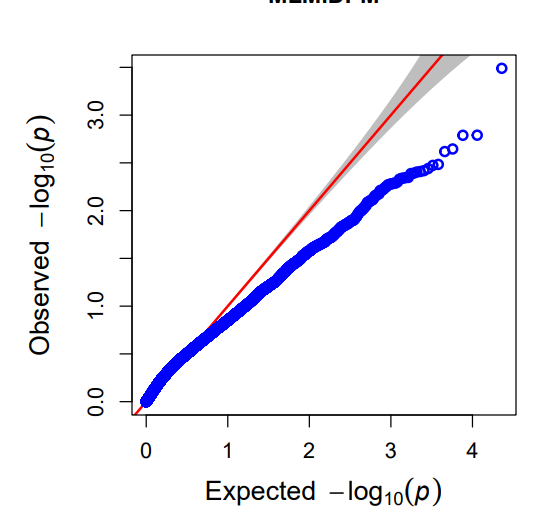


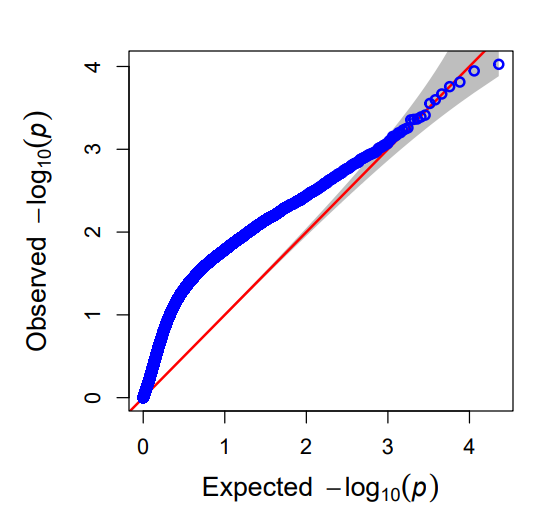


**C**   **D**


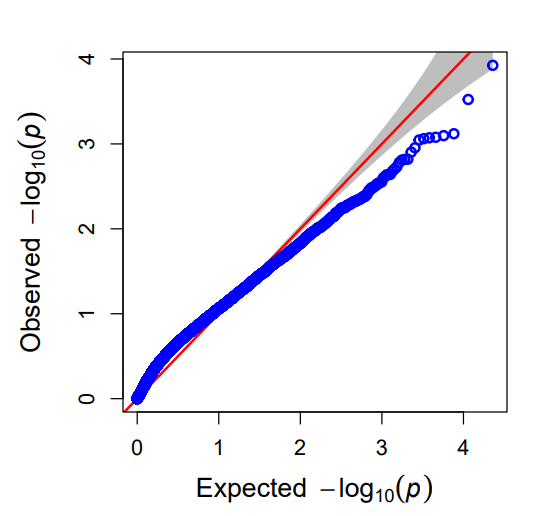


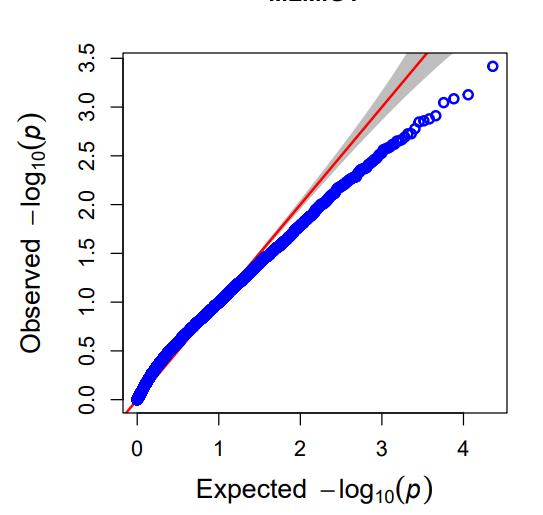


**E F**


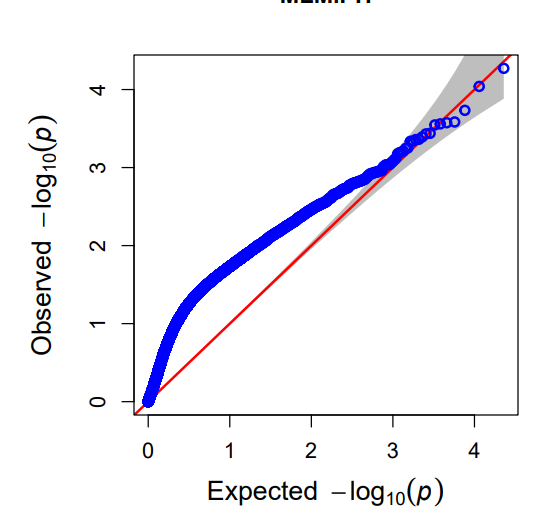


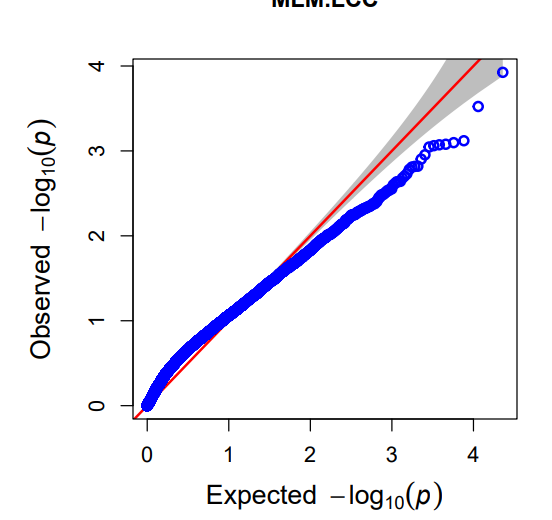


**S6 Fig. Quantile –Quantile (QQ) plots of the p- values observed and the expected from the genome-wide association study under well-watered conditions.** Note A **=** Days to 50% flowering, B **=** Days to physiological maturity, C **=** Grain yield, D **=** Leaf chlorophyll content, E **=** Plant height, F **=** Leaf temperature.
